# The Potential Role of Probiotics in Protection against Influenza a Virus Infection in Mice

**DOI:** 10.3390/foods10040902

**Published:** 2021-04-20

**Authors:** Wenwei Lu, Zhifeng Fang, Xinyang Liu, Lingzhi Li, Pinghu Zhang, Jianxin Zhao, Hao Zhang, Wei Chen

**Affiliations:** 1 State Key Laboratory of Food Science and Technology, Jiangnan University, Wuxi 214122, China; luwenwei@jiangnan.edu.cn (W.L.); zhifengf@jiangnan.edu.cn (Z.F.); elflxy@outlook.com (X.L.); 7150112014@vip.jiangnan.edu.cn (L.L.); zhaojianxin@jiangnan.edu.cn (J.Z.); zhanghao61@jiangnan.edu.cn (H.Z.); 2 School of Food Science and Technology, Jiangnan University, Wuxi 214122, China; 3 National Engineering Research Center for Functional Food, Jiangnan University, Wuxi 214122, China; 4 (Yangzhou) Institute of Food Biotechnology, Jiangnan University, Yangzhou 225004, China; 5 Institute of Translational Medicine & Jiangsu Key Laboratory of Integrated Traditional Chinese and Western Medicine for Prevention and Treatment of Senile Diseases, Medical College, Yangzhou University, Yangzhou 225009, China; zhangpinghu@yzu.edu.cn; 6 Wuxi Translational Medicine Research Center and Jiangsu Translational Medicine Research, Institute Wuxi Branch, Wuxi 214122, China

**Keywords:** influenza A virus, probiotic, immune response, gut microbiota, butyrate

## Abstract

Influenza A virus induces severe respiratory tract infection and results in a serious global health problem. Influenza infection disturbs the cross-talk connection between lung and gut. Probiotic treatment can inhibit influenza virus infection; however, the mechanism remains to be explored. The mice received *Lactobacillus mucosae* 1025, *Bifidobacterium breve* CCFM1026, and their mixture MIX for 19 days. Effects of probiotics on clinical symptoms, immune responses, and gut microbial alteration were evaluated. *L. mucosae* 1025 and MIX significantly reduced the loss of body weight, pathological symptoms, and viral loading. *B. breve* CCFM1026 significantly reduced the proportion of neutrophils and increased lymphocytes, the expressions of TLR7, MyD88, TRAF6, and TNF-α to restore the immune disorders. MIX increased the antiviral protein MxA expression, the relative abundances of *Lactobacillus*, *Mucispirillum*, *Adlercreutzia*, *Bifidobacterium*, and further regulated SCFA metabolism resulting in an enhancement of butyrate. The correlation analysis revealed that the butyrate was positively related to MxA expression (*p* < 0.001) but was negatively related to viral loading (*p* < 0.05). The results implied the possible antiviral mechanisms that MIX decreased viral loading and increased the antiviral protein MxA expression, which was closely associated with the increased butyrate production resulting from gut microbial alteration.

## 1. Introduction

Influenza A virus causes many types of host infections, including mammals such as pigs, whales, and humans, induces severe respiratory tract infection, and results in more than 500,000 annual deaths worldwide [1]. It has been attracted worldwide attention because of the rapid mutation, especially the high mortality and prevalence of animal-derived influenza virus. The influenza virus is mainly transmitted through aerosol and droplet [2]. Sugar chains containing sialic acid on the surface of the cell membrane is the receptor of the influenza virus, and it mediates the viral replication and reproduction in the cell [3]. The hemagglutinin of the viral membrane binds to the receptor on the respiratory mucosal epithelial cells and leads to the typical symptoms of infection including fever, headache, fatigue, and anorexia. These symptoms are closely associated with immune responses including innate immunity and adaptive immunity.

Previous studies have found that there is a bidirectional connection between the lung and gut and thus establish the lung–gut axis [4]. Gut dysbiosis affects the respiratory inflammation and results in diseases including allergic asthma and idiopathic pneumonia syndrome [5]. The gut and lung are parts of the mucosal immune system and act as a shared immunological interface [6,7]. Therefore, immune dysregulation of gut microbiota may affect the immune responses in the lung. Treatment with intestinally localized antibiotics is closely associated with lung inflammatory markers and histologic symptoms of infiltratory cells in ischemia reperfusion-induced lung inflammation [8]. This suggests that gut microbiota strongly influences immune responses in the lung and potentially be a target to alleviate the pulmonary inflammatory response. Furthermore, gut microbiota increases the lung defense against bacterial challenge through Toll-like 4 receptors signaling [9]. Collectively, gut microbiota plays a critical role in the intestinal mucosal immunity and its homeostasis is important for the crosstalk of lung–gut axis. Influenza A virus-induced respiratory infection disturbs the balance between the lung and gut bacteria and affects their cross-talk connection. Alteration of the gut microbiota in patients with coronavirus disease 2019 (COVID-19) or H1N1 influenza has been explored, suggesting that the intestinal microbiota dysbiosis might be associated with COVID-19 infection or H1N1 influenza [10]. Therefore, re-establishing gut microbial balance may contribute to the alleviation of patients with influenza A infection.

To regulate gut microbial balance, many approaches, such as prebiotic supplement and probiotic treatment, have been applied to both animal experiments and clinical trials [11,12]. Particularly, probiotics, severing as a common gut microbial regulator, affect the gut microbial diversity, structure, and composition. Probiotic treatment is able to alter the metabolic activity of gut microbiota and the metabolites including short-chain fatty acids (SCFA), are closely associated with the host immune response [13,14]. Butyrate is the major energy for proliferation and differentiation of colonocytes and exerts its anti-inflammatory effects via suppressing production of proinflammatory molecules such as TNF-α, IL-1β, and NF-κB and increasing IL-10 production [15]. Additionally, a study has reported that *Bifidobacterium longum* 35624^®^ significantly reduces viral load within the lung and improves the survival of mice via reducing IL-6 and type 1 and 2 interferon (IFN) levels and increasing IFN-λ and surfactant protein D [16]. Therefore, the probiotic treatment also affects the correlations related to immune response and pathological indicators between gut and lung. However, the mechanism varies with different probiotic strains on alleviating influenza virus infection. Heat-killed *Lactobacillus plantarum* L-137 decreases the viral titers in the lung and increases the survival time of mice after influenza virus A/FM/1/47 (H1N1) infection [17]. *B. longum* BB536 treatment reduces the loss of body weight and viral proliferation and decreases the interleukin-6 (IL-6) and interferon-γ (IFN-γ) levels to alleviate symptoms of influenza virus infection [18]. Therefore, combining with the changes in gut microbiota, the antiviral characters of different probiotics need to be further explored.

In this study, influenza A virus A/FM1/47 (H1N1) was used to induce respiratory infection in a mouse model. This model was used to evaluate the effects of *Lactobacillus mucosae* DL3-9 (1025) and *Bifidobacterium breve* GuXi-2016-6-7 (CCFM1026) on the clinical symptoms, immune responses, and gut microbial alteration. To further reveal the mechanism of probiotics on alleviating influenza virus infection, the gut microbial SCFA metabolism was measured and established the correlation with the disease indicators.

## 2. Materials and Methods

### 2.1. Bacterial Strains

*L. mucosae* 1025 and *B. breve* CCFM1026 were stored at the Culture Collection of Food Microorganisms (CCFM) in Jiangnan University (Wuxi, Jiangsu, China). *L. mucosae* 1025 was cultured in de Man, Rogosa and Sharpe (MRS) broth (Beijing Solarbio Science & Technology Co., Ltd., Beijing, China) at 37 °C for 16 h. *B. breve* CCFM1026 was cultured in MRS broth with 0.05% (*w*/*v*) L-cysteine-HCl (Sinopharm Chemical Reagent Co., Ltd., Shanghai, China) at 37 °C for 48 h in an anaerobic incubator (AW500SG, Electrotek England, Shipley, UK).

### 2.2. Animals and Treatment

**Figure 1:**
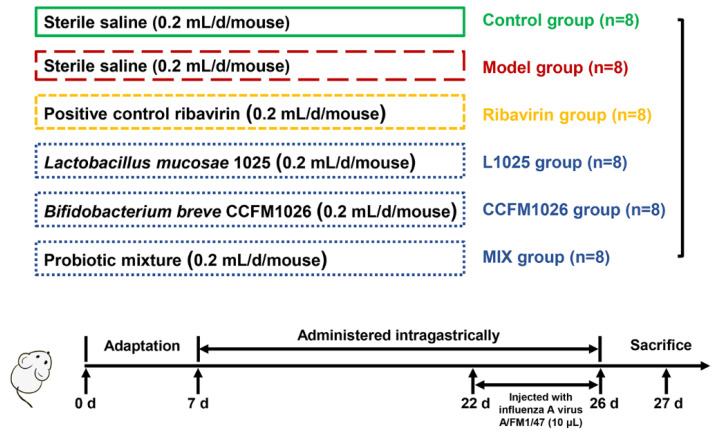
The flow of animal experiment.

ICR mice (3 week old, female) were purchased from the Comparative Animal Medicine Center of Yangzhou University (Yangzhou, Jiangsu, China) and kept in a facility with a controlled light cycle (12 h/12 h light/dark), temperature (25 ± 2 °C), and humidity level (50%). Mice were fed standard commercial chow and water ad libitum. The experiment lasted for 26 days. After 1 week adaptation, 48 mice were divided into the 6 groups (*n* = 8): control, model, positive control ribavirin (Sigma Aldrich Co., Ltd., St. Louis, MO, USA, Ribavirin), *L. mucosae* 1025 (L1025), *B. breve* CCFM1026 (CCFM1026), and the mixture (*L. mucosae* 1025: *B. breve* CCFM1026 = 1:1, MIX) (Figure 1). Except for the control group, all other mice were infected with a dose of 5-times the 50% lethal dose (5LD50) of the influenza A virus A/FM1/47 (H1N1), a mouse lung adaptive strain, which was provided by the Key Laboratory of Avian Infectious Diseases, Ministry of Agriculture, Yangzhou University. On day 22, after anesthetized, the mice were injected with 10 μL virus via nasal feeding for 5 days. The probiotic suspension of 0.2 mL (1 × 10^9^ CFU) was orally administrated for 19 days (after adaptation), and the number of living bacteria were determined using the plate counting method. The ribavirin group was orally administered with ribavirin after infection. The mice in the control and model group were received an equal volume (0.2 mL) of saline in the experimental period.

### 2.3. Change in Weight

On the days 22, 24, 25, 26, the weight of mice was measured for calculating the change.

### 2.4. Lung Histopathology

A pathological picture of the lung was collected using a digital camera (D750, Nikon Corp., Tokyo, Japan) at the day 27 (sacrifice). Lung samples were fixed and embedded using formalin and paraffin (Sinopharm Chemical Reagent Co., Ltd., Shanghai, China), respectively. The thickness of the lung slice was 5 μm and stained with hematoxylin and eosin (HE, Yulu Laboratory Equipment Co., Ltd., Nanchang, Jiangxi, China). Photomicrographs (original magnification = 400×) were obtained using a digital scanner (Pannoramic MIDI, 3DHistech Ltd., Budapest, Hungary).

### 2.5. Virus Loading, MxA, and Immune Indicators Measurement

The expressions of MxA, TLR7, MyD88, TRAF6, and TNF-α were determined using quantitative real-time PCR (qPCR). Changes in viral nuclear proteins (NP) was used to characterize virus loading. After sacrifice, the lung section was collected and homogenized with 0.01 M phosphate-buffered saline (pH 7.20) at 65 HZ for 10 min. RNA was extracted from the lung tissues using TRIzol reagent (Invitrogen Life Technologies, Carlsbad, CA, USA) according to the manufacturer’s instruction. The qPCR was performed using iTaq Master SYBR Green Super Mix (Bio-Rad, Hercules, CA, USA) in a RT-PCR system (Thermal cycler CFX96, Bio-Rad, Hercules, CA, USA). The relative expression of genes was normalized to that of GAPDH and calculated according to the 2^−ΔΔCT^ approach. Primers used are shown in Table 1.

### 2.6. Blood Cell Analysis

After sacrifice, blood samples of mice were collected in the anticoagulation tube with EDTA-K2 at room temperature. After mixing, blood cell analysis (50 μL sample) was performed using an automatic hematology analyzer (BC-5000 vet, Shenzhen Mindray Biomedical Electronics Co., Ltd., Shenzhen, China) to reveal the alteration in the proportion of lymphocytes and neutrophils.

### 2.7. Gut Microbial Profiling

DNA from the feces was obtained using a FastDNA spin kit for feces (MP Biomedicals, Santa Ana, CA, USA) according to the manufacturer’s instructions. The V3-V4 region of the 16S rRNA gene was amplified (341F and 806R) and sequenced using the Illumina sequencing platform (MiSeq, Illumina Co., Santiago Canyon, CA, USA). Briefly, PCR products were excised from a 2.0% agarose gel (Sangon Biotech, Sangon Biotech (Shanghai) Co., Ltd., Shanghai, China) and purified using TIANgel mini purification kit (Tiangen, Tiangen Biotech (Beijing) Co., Ltd., Beijing, China). DNA concentration was measured using the Qubit BR dsDNA assay. Libraries were prepared using TruSeq DNA LT sample preparation kits (Illumina) and sequenced for 500 + 7 cycles on the MiSeq platform (Illumina) using the MiSeq reagent kit (Illumina). 16S rRNA sequence data were measured using the QIIME pipeline. The raw sequences were screened (low-quality (score < 30) and short length (<200 bp) sequences were abandoned) and the qualifying sequences were spliced. Sequences with similarity >97% were clustered into operational taxonomic units (OTU) and representative sequences of each cluster were used to classify bacterial taxa.

### 2.8. Change in SCFA Metabolism

SCFA concentrations were calculated using the external standard method and measured using gas chromatography-mass spectrometry (GCMS-QP2010 Ultra, Shimadzu Corp., Kyoto, Japan) referring to the previous study [19]. Briefly, colonic contents (25–50 mg) were mixed with a 500 μL saturated NaCl solution (Sinopharm) for 30 min. Then, the samples were acidified with 40 μL of 10% sulfuric acid (Sinopharm), and 1000 μL ether (Sinopharm) was added for SCFA extraction. The mixture was centrifuged at 12,000× *g* for 15 min at 4 °C (5424, Eppendorf Co., Hamburg, Germany). The supernatant was transferred to the gas-phase vial for GC-MS analysis. The injection temperature was 240 °C, 1 μL prepared sample was injected and separated on a Rtx-Wax column (30 m × 0.25 mm (internal diameter), 0.25 μm, Shimadzu) with helium as the carrier gas (flow rate: 2 mL/min, split ratio: 10:1). The GC temperature program was as follows: a temperature ramp from 100 to 140 °C at the rate of 7.5 °C/min and increased to 200 °C by 60 °C/min, and then the temperature was maintained for 3 min. The ionization temperature was 220 °C. The standards of acetic acid, propionic acid, and butyric acid (Sigma-Aldrich) were mixed and used at different concentrations and measured with the same conditions. The peak of each sample was compared to the same standard peak to obtain the concentration of each of the SCFA.

### 2.9. Statistical Analysis

The statistical analyses were processed using GraphPad Prism 8 (GraphPad Inc., La Jolla, CA, USA). Data are shown as the mean ± SD. The network correlation between variations was done using R (version 3.5.1, corrplot package). *p* < 0.05 was considered statistically significant.

## 3. Results

### 3.1. Probiotic Mixture Suppressed the Loss of Body Weight

**Figure 2:**
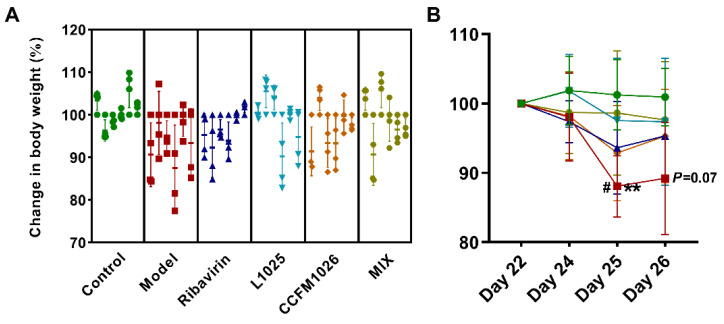
Change in body weight of mice (**A**). Changes in body weight of every mouse (**B**). Changes in weight of mice in each group, and statistical analysis for the model group: **, day 25 vs. day 22; #, day 25 vs. day 24; and *p* = 0.07, day 26 vs. day 22 (two-way ANOVA and Tukey’s multiple comparisons).

To explore the effects of the virus on the body weight of mice during probiotic treatments, the weight change was measured on days 22, 24, 25, and 26 (Figure 2). The weight of all mice was decreased after the virus treatment in the model group, but it kept stable in the control and MIX groups (Figure 2A). The weight of mice was increased on day 24 but decreased on day 25 and then kept stable in the L1025 group (Figure 2B). However, *B. breve* CCFM1026 could not reduce the sustained weight loss. On days 25 and 26, the weight was significantly lower (88.1% ± 4.40% and 89.2% ± 8.08%, respectively) than that on day 22 (100%) in the model group (Figure 2B). The weight loss occurred on day 25 in the model (88.1% ± 4.40%), ribavirin, L1025, and CCFM1026 groups, and particularly, the weight of mice was significantly lower than that on day 24 in the model group (98.1% ± 6.34%). The results showed that *L. mucosae* 1025 and the probiotic mixture had the potential to prevent and suppress weight loss caused by virus infection.

### 3.2. Probiotic Mixture Improved the Pathological Features of Lung

**Figure 3:**
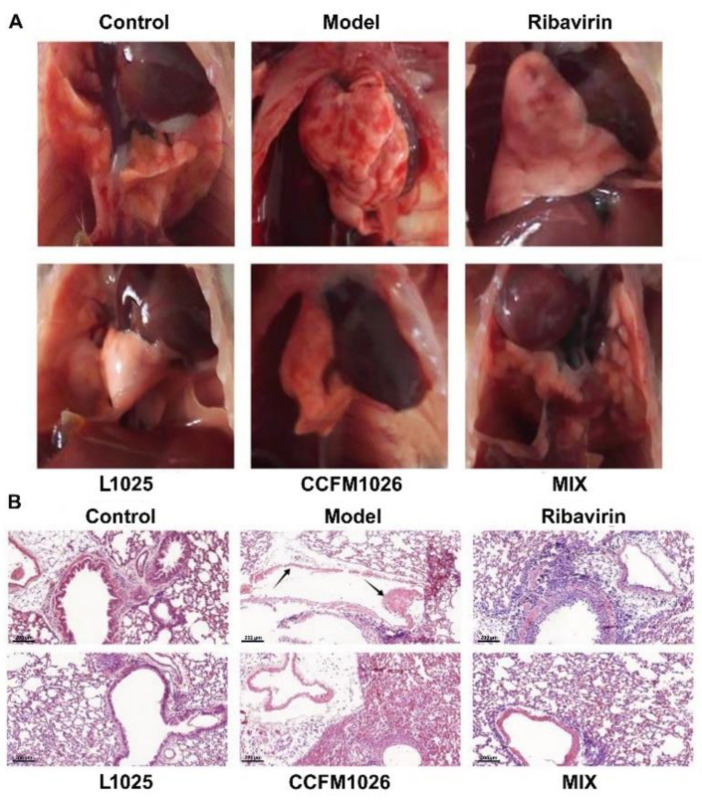
Pathological features of lung. (**A**) Pathological pictures of the lung. (**B**) HE (hematoxylin and eosin) staining for the lung sections; the arrows indicate inflammatory infiltration and abruption of the bronchial epithelium. Scale bar = 200 μm, original magnification = ×400.

The influenza virus infection commonly causes inflammation in the lung. Therefore, to reveal the effects of probiotic strains on the pathological symptoms of lung, the pathological picture and HE staining of the lung were performed (Figure 3). After the virus infection, there was severe inflammation in the lung of mice in the model group and the collapsed structure led to the lung atrophy (Figure 3A). On the contrary, the lung structure of mice was intact and had no inflammatory infiltration in the control group. Ribavirin treatment suppressed the inflammation and maintained the integrity of the lung. Probiotic groups (L1025, CCFM1026, and MIX) were similar to the ribavirin group, and they had the potential to improve the lung pathological features. HE staining showed that there was no inflammation on the trachea and bronchus and the alveoli were intact in the control group (Figure 3B). While in the model group, the structure of the trachea was seriously damaged (black arrow) and there were no intact alveoli, *L. mucosae* 1025 and MIX treatments significantly suppressed the inflammation on the trachea and restored the integrity of alveoli. *B. breve* CCFM1026 treatment maintained the integrity of the trachea and had alleviating effects on the lung pathological symptoms, although there was inflammatory infiltration in the lung.

### 3.3. Probiotic Strains Regulated Systemic Immune Responses

**Figure 4:**
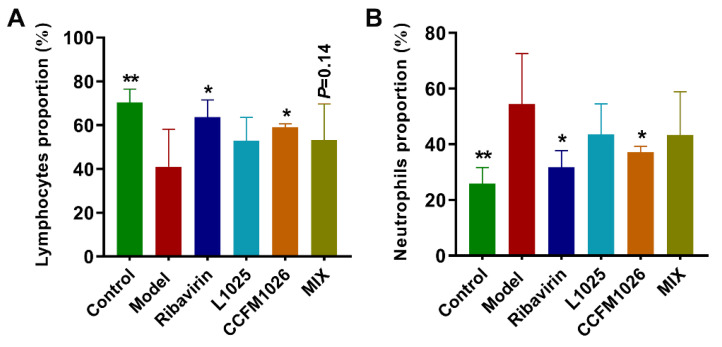
Alteration in systemic immune responses. (**A**) The proportion of lymphocytes. (**B**) The proportion of neutrophils. The statistical difference was evaluated using one-way ANOVA and post hoc Fisher’s least significant difference (LSD) tests (*, ** vs. the model group).

To explore the effects of probiotic strains on inflammatory responses, alteration in the proportion of lymphocytes and neutrophils in serum was determined. Compared to the control group (70.4% ± 6.05%), the number of lymphocytes was decreased after influenza virus infection in the model group (41.0% ± 17.1%), but they were significantly restored using ribavirin (63.7% ± 7.85%) and *B. breve* CCFM1026 (59.1% ± 1.52%) treatments (Figure 4A). *L. mucosae* 1025 and MIX treatments increased the proportion of lymphocytes although there was no statistical significance. The proportion of neutrophils increased in the model group (54.4% ± 18.2%) versus the control group (25.9% ± 5.84%) (Figure 4B). Ribavirin (31.7% ± 5.91%) and *B. breve* CCFM1026 (37.1% ± 2.19%) treatments significantly reduced the number of neutrophils versus the model group. However, *L. mucosae* 1025 and MIX could not significantly suppress the increase in the proportion of neutrophils. The results demonstrated that probiotic treatments improved virus infection-induced inflammatory responses in mice.

### 3.4. Probiotic Strains Affected the Antiviral Signaling Pathway

**Figure 5:**
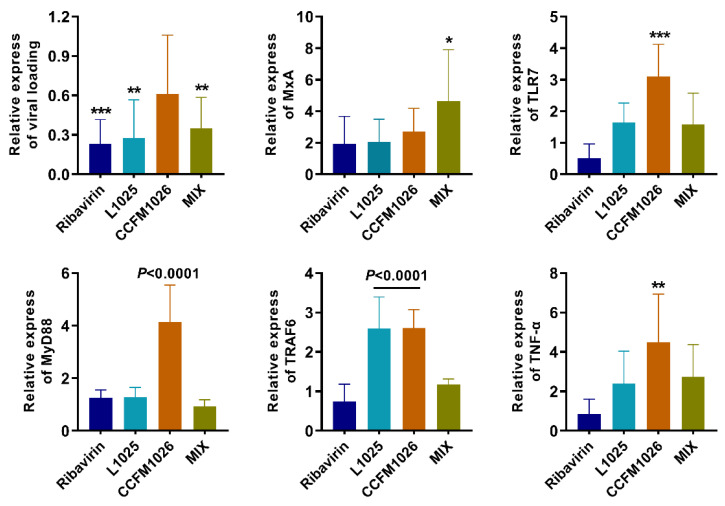
Alteration in indicators related to the antiviral signaling pathway. The statistical difference was evaluated using one-way ANOVA and Dunnett’s multiple comparisons test (*, **, ***, *p* < 0.0001 vs. the model group).

To explore the effects of probiotic strains on the virus and antiviral indicator, expression of the viral loading and an antiviral protein MxA were measured. Compared to the model group, *L. mucosae* 1025 and MIX treatments significantly decreased the viral loading but CCFM1026 could not (Figure 5). *L. mucosae* 1025 and *B. breve* CCFM1026 did not affect MxA expression. However, MIX treatment significantly increased the MxA expression. The results showed that the mechanisms for alleviating influenza virus infection were different between these probiotic strains and the mixture MIX had the more potential to defense and clear the virus in the host. Therefore, we next determined the expression of indicators in the antiviral signaling pathway TLR7-MyD88-TRAF6. *B. breve* CCFM1026 significantly increased the expressions of TLR7, MyD88, TRAF6, and TNF-α versus the model group and affected the signaling pathway (Figure 5). *L. mucosae* 1025 significantly increased the TRAF6 levels but did not affect other indicators in this pathway. However, there was no effect of MIX on the expressions of these indicators. These results provided further evidence that probiotic strains exerted the strain-specific alleviating effect on influenza virus infection and related to different mechanisms for defending against influenza infection.

### 3.5. Probiotic Strains Altered the Gut Microbial Composition

#### 3.5.1. Changes in the Phylum Level

**Figure 6:**
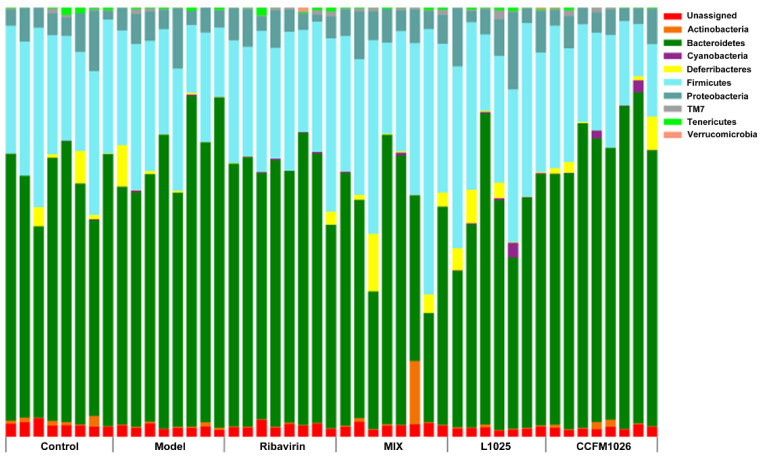
Changes in gut microbiota at the phylum level.

To explore the effects of probiotics on gut microbiota at the phylum level 16S rRNA amplification sequencing analysis was performed. At the phylum level, *Bacteroidetes*, *Firmicutes*, and *Proteobacteria* were the major components in all groups, and particularly, *Deferribacteres* was one of the important constituents in all groups except for the model group (Figure 6). Compared to the control group, the proportion of *Bacteroidetes* was increased but *Firmicutes* and *Deferribacteres* were decreased after influenza virus infection in the model group (*Firmicutes*/*Bacteroidetes* ratio (F/B) = 0.38). *L. mucosae* 1025 restored the relative abundances of *Firmicutes* and *Deferribacteres* and increased *Firmicutes* versus the model group. However, *B. breve* CCFM1026 increased the proportion of *Bacteroidetes* and decreased *Firmicutes*, and the ratio of *Firmicutes*/*Bacteroidetes* was 0.32. The structure and composition of gut microbiota in the *B. breve* CCFM1026 group were similar to the model group. The effects on gut microbial alteration were different between *L. mucosae* 1025 and *B. breve* CCFM1026 and this might be one of the reasons for the difference in alleviating mechanisms between the two strains. MIX treatment significantly decreased the proportion of *Bacteroidetes* but increased *Firmicutes* and *Deferribacteres* and restored the gut microbial dysbiosis. The results showed that probiotic treatments significantly affected the gut microbial composition and metabolic functions contributing to the difference in alleviation of infection.

#### 3.5.2. Clustering Analysis at the Genus Level

**Figure 7:**
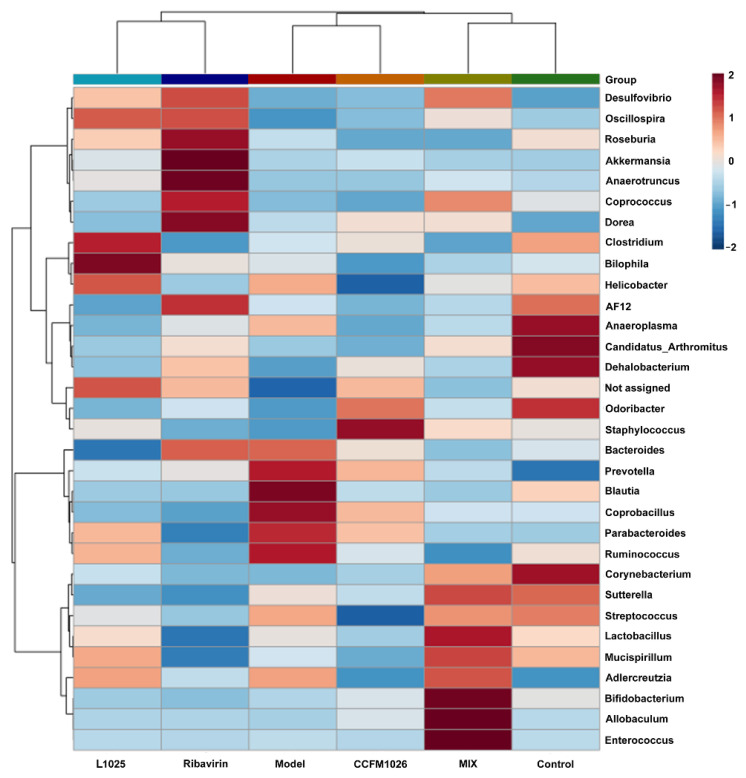
Heatmap related to clustering analysis and alteration in gut microbiota.

To reveal the altered gut microbiota at the genus level, a heatmap related to clustering analysis was established. The heatmap showed that the MIX and control groups, L1025 and ribavirin groups, and CCFM1026 and model groups were clustered together on a branch (Figure 7). Overall, there were similarities in the composition at the genus level between the three clustered groups. In the model group, the relative abundances of *Bacteroides*, *Prevotella*, *Blautia*, *Coprobacillus*, *Parabacteroides*, and *Ruminococcus* were increased but they were decreased in the control group. *Anaeroplasma*, *Candidatus Arthromitus*, *Dehalobacterium*, *Odoribacter*, and *Corynebacterium* were increased in the control group. *L. mucosae* 1025 treatment increased the proportion of *Oscillospira*, *Clostridium*, *Bilophila*, and *Helicobacter* but *B. breve* treatment increased *Odoribacter* and *Staphylococcus*. MIX treatment significantly affected the gut microbial composition and this was consistent with the changes of microorganism at the phylum level. There were increases in 9 genera including *Lactobacillus*, *Bifidobacterium*, *Allobaculum*, *Desulfovibrio*, *Coprococcus*, *Sutterella*, *Mucispirillum*, *Adlercreutzia*, and *Enterococcus* in the MIX group. The results showed that probiotic strains exerted different effects on alteration in gut microbial structure and composition and thus had their unique mechanism of actions on preventing influenza infection.

### 3.6. Probiotic Strains Affected SCFA Production and the Correlation with Disease Indicators

**Figure 8:**
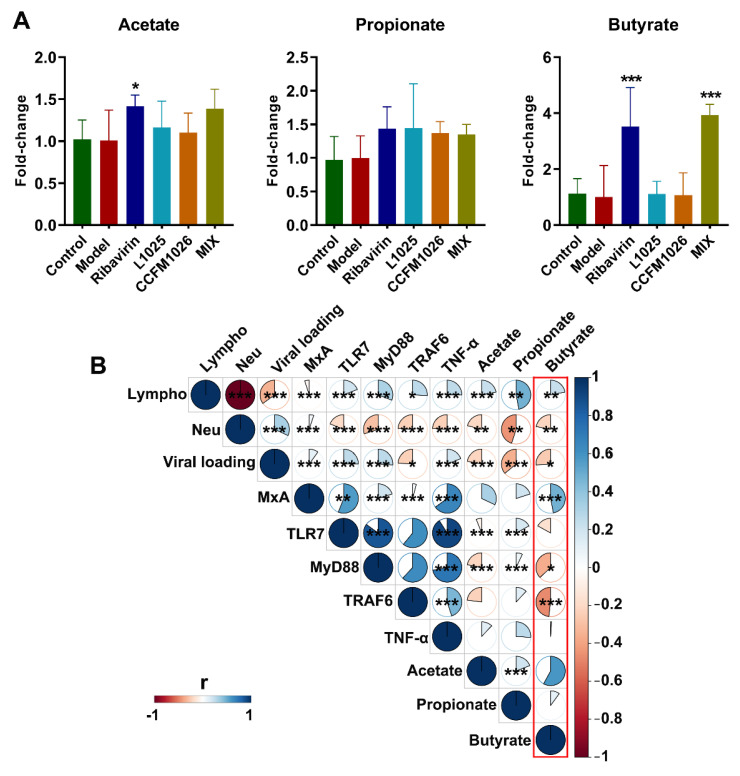
Short-chain fatty acids (SCFA) production and the correlation with disease indicators. (**A**) Fold-change in SCFA (the statistical difference was evaluated using one-way ANOVA and post hoc Fisher’s least significant difference (LSD) tests, *, *** vs. the model group). (**B**) The correlation between SCFA and disease indicators. * *p* < 0.05, ** *p* < 0.01 and *** *p* < 0.001.

Probiotic treatments altered gut microbial composition and thus affected their metabolic activities including SCFA metabolism. To determine the alteration in SCFA, the concentrations of acetate, propionate, and butyrate were measured. Compared to the model group, *L. mucosae* 1025, *B. breve* CCFM1026, and MIX treatments increased the concentration of acetate and propionate although there was no statistical significance (Figure 8A). *L. mucosae* 1025 and *B. breve* CCFM1026 could not affect the butyrate production, but MIX treatment (3.9 ± 0.39) significantly increased the concentration of butyrate versus the model group. The results showed that probiotic treatments altered the metabolism of gut microbiota, and particularly, the mixture of *L. mucosae* 1025 and *B. breve* CCFM1026 significantly elevated the concentration of butyrate.

To evaluate the effects of SCFA on immune responses in influenza infection, the correlation analysis between SCFA and disease indicators was performed. The butyrate was positively related to lymphocytes proportion and MxA expression (*p* < 0.001) but was negatively related to neutrophils proportion, viral loading, MyD88, and TRAF6 expressions (*p* < 0.001) (Figure 8B, red frame). The correlation between acetate and disease indicators was similar to butyrate but could not be significantly related to MxA and TRAF6 expressions (*p* > 0.05). Propionate was negatively related to neutrophils proportion and viral loading but positively related to TLR7 and MyD88 expressions (*p* < 0.001). The correlations between acetate, propionate, and butyrate and immune responses were distinctive, although *L. mucosae* 1025, *B. breve* CCFM1026, and MIX treatment could not significantly regulate acetate and propionate production. According to the correlation analysis, these differences of probiotic strains in metabolism might lead to the strain-specific effects on alleviating influenza infection.

## 4. Discussion

It has been reported that probiotic with regulating the balance of intestinal microecology exerts prophylactic and alleviating effects on diseases, such as allergic asthma [20,21], atopic dermatitis [22,23], and influenza infection [24,25], but the correlation between clinical characters of influenza infection and gut microbial alteration needs to be explained. Based on the gut–lung axis theory, the bi-directional cross-talk between lung and gut intricately influences the homeostasis of both [26], and therefore, the deeper understanding of gut microbial alteration on alleviating respiratory disorders contributes to therapeutic applications using probiotic strains. Here, the effects of *L. mucosae* 1025, *B. breve* CCFM1026, and their mixture MIX on infection of influenza A virus strain A/FM1/47H1N1 were evaluated by physiological alterations, including bodyweight loss, pathological changes in lung, gut microbial changes, and SCFA production. Additionally, the potential mechanisms of *L. mucosae* 1025, *B. breve* CCFM1026, and MIX were further explored. *L. mucosae* 1025, *B. breve* CCFM1026, and MIX improved the clinical symptoms of respiratory infection but the alleviating mechanism was distinctive.

Influenza infection causes the loss of body weight, and it seems to be a surrogate marker of disease severity after influenza virus infection [27]. Therefore, body weight loss was evaluated after influenza virus infection during probiotic treatments. The results showed that in the model group, mice infected with virus revealed a significant body weight loss on the day 25 (11.9%) versus the initial weight (Figure 2B). *L. mucosae* 1025 and MIX treatments significantly prevented the loss of body weight (weight loss: 2.5% and 1.4%, respectively) after virus infection. However, *B. breve* CCFM1026 (92.9% ± 6.85%, day 25) was similar to the positive control drug ribavirin (93.6 ± 6.66%, day 25) and had weak effects on resisting in a reduction of body weight loss. Interestingly, although *B. breve* CCFM1026 had weak effects on maintaining body weight, the mixture (MIX) of CCFM1026 and *L. mucosae* 1025 exerted a commendable performance on suppressing reduction for weight loss even better than *L. mucosae* 1025 used independently. This synergistic effect of the mixture showed that there was a distinctive mechanism to alleviate influenza infection. The histopathological observation of the lung showed that influenza A virus-induced severe inflammation led to the collapsed structure versus the integrated structure in the control group. However, *L. mucosae* 1025, *B. breve* CCFM1026, and MIX treatments reversed the inflammatory infiltration of the lung after influenza A virus infection. The pathological symptoms of the lung in three probiotic groups were significantly improved but had a few inflammatory infiltrations, and similar to those in the positive control ribavirin group (Figure 3A). HE staining further confirmed the alleviating effects of probiotic strains (Figure 3B). The consumption of probiotic cocktail (*L. gasseri* PA 16/8, *B. longum* SP 07/3, and *B. bifidum* MF 20/5) significantly decreased the symptom score, the duration of episodes, and the days with fever in a clinical trial [28,29]. The probiotic cocktail increased the proportions of cytotoxic T lymphocyte (CD8+) and T helper cells (CD4+) and regulated the immune responses. Additionally, it increased the relative abundance of lactobacilli and bifidobacteria and altered the gut microbial structure and composition. These results implied that the probiotic-induced improvement in the influenza virus was closely associated with immune responses and gut microbial alteration.

Lymphocytes are an important component and the main effector cell of immune function in the lymphatic system [30]. They play crucial roles in asthma, tissue repair, and responses to helminths [31]. Neutrophils have been considered as the final effector cells responding to acute inflammation, with the role of eliminating pathogens [32]. Therefore, changes in lymphocytes and neutrophils are important for diseases including infections caused by pathogens. An increase in the proportion of neutrophils was responded to influenza A virus infection in the model group, while, correspondingly, there was a decrease in lymphocytes (Figure 4). This revealed that there were stress and inflammatory responses after infection in the model group. *B. breve* CCFM1026 significantly regulated the inflammatory responses but *L. mucosae* 1025 and MIX had weak effects on alteration in lymphocytes and neutrophils. This implied that *B. breve* CCFM1026 might derive the antiviral effect through the inflammatory regulation. However, antiviral effects of *L. mucosae* 1025 and MIX were associated with other factors such as viral loading and antiviral protein. Therefore, we determined the relative expressions of viral loading and an antiviral protein MxA. *L. mucosae* 1025 and MIX significantly decreased the relative expression of viral loading and MIX increased MxA (Figure 5). However, *B. breve* CCFM1026 could not alter the relative expression of both. Obviously, *L. mucosae* 1025 alleviated the clinical symptoms of the lung by directly decreasing viral loading. While MIX not only reduced the expression of viral loading but increased the MxA to restore the pathological characters-induced by influenza A virus infection.

Toll-like receptors (TLR) are a class of pattern recognition receptors that detect pathogen-associated molecular patterns [33]. It has been reported that TLR ligands such as TLR4 and TLR7 have been employed to increase immunogenicity against influenza virus infection [34]. Influenza A virus activated-TLR7, and the adaptor protein MyD88 increased the expression of protective cytokines including type I interferons (IFN), interleukin (IL)-6, and IL-1β [35]. Probiotic treatment significantly upregulates the expressions of TLR7, MyD88, IRAK4, TRAF6, and NF-κB to alleviate FM1 influenza virus-induced respiratory tract infection [36]. This reveals that the TLR7 signaling pathway plays a key role in the regulation of respiratory influenza virus infection. We determined whether *L. mucosae* 1025, *B. breve* CCFM1026, and MIX affect the TLR7 signaling pathway in influenza A virus-respiratory infection. The results showed that *B. breve* CCFM1026 significantly increased the mRNA expression of TLR7, MyD88, TRAF6, and TNF-α but *L. mucosae* 1025 and MIX could not activate the TLR7 signaling pathway (Figure 5). TNF, one of CD8 T cell effectors, maybe not essential for suppressing viral replication [37]. However, some studies have been reported that TNF-α inhibits the replication of viruses such as influenza and hepatitis B virus and serves as the first line of defense against influenza virus infection [38,39]. Therefore, we deduced that *B. breve* CCFM1026 regulated the antiviral responses by activating the TLR7 signaling pathway and increasing the expression of TNF-α. This deduction was also coincident with the above results of immune regulation.

Emerging evidence shows that the balance and metabolites of gut microbiota contribute to the healthy homeostasis of the immune system [40]. Some studies have been reported that alteration in gut microbiota is closely associated with diverse diseases such as inflammatory bowel disease [41], nonalcoholic fatty liver disease [42], and respiratory infection [43]. Gut microbiota affects the onset and development of respiratory infection through the gut–lung axis. Disturbance of gut microbiota reduced the antiviral responses and thus leads to severe clinical symptoms. Therefore, the gut microbial composition was evaluated after virus infection. The virus-induced low F/B ratio was significantly restored by *L. mucosae* 1025 and MIX treatments but *B. breve* CCFM1026 could not increase the F/B ratio (Figure 6). Changes in microbiota at the genus level further revealed that the structure of the *B. breve* CCFM1026 group was similar to that in the model group (Figure 7). This revealed that *B. breve* CCFM1026-produced improvement might be not associated with gut microbiota. However, MIX treatment increased the relative abundances of beneficial bacteria such as *Lactobacillus*, *Mucispirillum*, and *Bifidobacterium*, and the overall microbial structure was similar to that in the control group (Figure 7). *Mucispirillum*, the sole known representative of *Deferribacteres* present in the mammalian microbiota, antagonizes *Salmonella* to protect mice against colitis [44]. Several studies have been demonstrated that *Lactobacillus* and *Bifidobacterium* are beneficial for nutrient absorption and significantly affect human health and disease. The increases in these beneficial bacteria help not only restore homeostasis of gut microbiota but also regulate the immune responses related to the gut–lung axis. *L. mucosae* 1025 and ribavirin groups were clustered to a branch and indicated that they were similar in the structure. These gut microbial alterations led to differences in metabolism. SCFA, the common gut microbial metabolite, is closely associated with host immune regulation [45]. Fecal transfer experiments have been demonstrated that gut dysbiosis-induced altered SCFA production contributes to pneumococcal infection by affecting the immunoactivity of alveolar macrophages during influenza episodes [46]. *L. mucosae* 1025 and *B. breve* CCFM1026 could not significantly alter acetate, propionate, and butyrate production versus the model group (Figure 8A). However, MIX treatment significantly increased butyrate concentration. The results suggested MIX treatment-altered butyrate might contribute to the alleviation of clinical symptoms after influenza A virus infection.

To explore the connection between SCFA, particularly, altered butyrate and disease indicators, the correlation analysis was performed. Acetate, propionate, and butyrate positively related to lymphocytes proportion and negatively related to neutrophils and viral loading although all probiotic treatments could not significantly affect acetate and propionate production (Figure 8B). Additionally, butyrate positively related to MxA expression (*p* < 0.001) and negatively related to MyD88 and TRAF6. The results suggested that altered SCFA had the potential to regulate host immune responses. MIX treatment significantly increased the MxA expression and decreased the viral loading (Figure 5) and this implied that altered MxA expression and viral loading were closely associated with increased butyrate production. In mechanistic terms, butyrate production resulting from beneficial bacteria affected by MIX treatment might regulate antiviral protein MxA expression and viral loading and thus alleviated influenza A virus-induced clinical symptoms. Although *L. mucosae* 1025 and *B. breve* CCFM1026 treatments altered the gut microbial composition, they might mediate the antiviral effects by directly reducing viral loading or immunoregulation. Therefore, probiotics exerted strain-specific effects on the alleviation of the influenza A virus.

## 5. Conclusions

In summary, the results showed that the clinical symptoms were improved by probiotic treatments and exerted different mechanisms with varying probiotic strains. *L. mucosae* 1025 directly reduced viral loading in the lung, and *B. breve* CCFM1026 might regulate the immune responses. However, their mixture MIX decreased viral loading and increased the antiviral protein MxA expression, which was closely associated with the increased butyrate production resulting from gut microbial alteration. This suggested that alteration in gut microbiota played a crucial role in cross-talk of the gut–lung axis.

## Figures and Tables

**Table 1:** Information for primer sequences.

Primers	Forward/Reverse	Sequence (5′ to 3′)
GAPDH	Forward	AGAGTGGGAGTTGCTGTTG
	Reverse	GCCTTCCGTGTTCCTACC
NP	Forward	GGCACCAAACGGTCTTACGA
	Reverse	TCACCTGATCAACTCCATTACCA
MxA	Forward	CCAACTGGAATCCTCCTGGAA
	Reverse	GCCGCACCTTCTCCTCATAG
TLR7	Forward	GATCGTGGACTGCACAGACA
	Reverse	CAGATGGTTCAGCCTACGGA
MyD88	Forward	ACTTGTTAGACCGTGAGGAT
	Reverse	CTCGGACTCCTGGTTCTG
TRAF6	Forward	TCTGCTTGATGGCTTTACG
	Reverse	ACCGTCAGGGAAAGAATCT
TNF-α	Forward	GGGCTACAGGCTTGTCACTCG
	Reverse	ACTCCAGGCGGTGCCTATGTC

## Data Availability

Data sharing not applicable.
